# The Integration of Macroscopic Tumor Invasion of Adjacent Organs into TNM Staging System for Colorectal Cancer

**DOI:** 10.1371/journal.pone.0052269

**Published:** 2012-12-26

**Authors:** Ji-wang Liang, Peng Gao, Zhen-ning Wang, Yong-xi Song, Ying-ying Xu, Mei-xian Wang, Yu-lan Dong, Hui-mian Xu

**Affiliations:** 1 Department of Surgical Oncology and General Surgery, First Hospital of China Medical University, Shenyang, People’s Republic of China; 2 Department of Tumor Pathology and Surgical Oncology, First Hospital of China Medical University, Shenyang, People’s Republic of China; B.C. Cancer Agency, Canada

## Abstract

**Objective:**

In addition to pathological TNM (pTNM) staging, the macroscopic staging (surgical TNM, sTNM) is another method used to stage and assess tumors, and it also potentially influences patient treatment guidelines. However, for the same patient, surgeons and pathologists might assess tumor depth differently. We aimed to evaluate the prognosis of patients who exhibit unconformity of intraoperative and postoperative results and propose a revised pT category (r-pT category) to predict survival in colorectal cancer.

**Methods and Results:**

In our study, 948 colorectal cancer patients were reviewed. We proposed a novel r-pT category in which surgical macroscopic T4b (sT4b) is incorporated into the pT category, namely, patients in the pT3 category with sT4b cancers are reclassified as being in the r-pT4a category; patients in the pT4a category with sT4b cancers are reclassified as being in the r-pT4b category. Cancer-specific survival according to the r-pT category was analyzed using Kaplan-Meier survival curves. A two-step multivariate analysis was used to determine correlations between the r-pT category and the prognosis. Harrell’s C statistic was utilized to test the predictive capacity. There were significant prognostic differences among the r-pT subcategories. We substituted the r-pT category for the pT category in current TNM staging in a 2-step multivariate analysis. The Harrell’s C statistical analysis results demonstrated that the r-pT category had superior predictive capacity compared to the pT category (Harrell’ C: 0.668 vs. 0.636; P = 0.002).

**Conclusions:**

Patients in the pT3 category with sT4b cancers, and patients in the pT4a category with sT4b cancers, are potentially under-staged, reclassification into higher categories could potentially benefit these patients. The results indicate that the r-pT category we proposed is potentially superior to the pT category in the assessment of prognosis for colorectal cancer.

## Introduction

Colorectal cancer is the third most common malignancy in the Western world, as well as the third leading cause of cancer-related death worldwide [1]. In China, the incidence of colorectal cancer is gradually increasing annually [2]. The International Union Against Cancer (UICC)/American Joint Committee on Cancer (AJCC) TNM staging system has been used for the staging of colorectal cancer for many years. The TNM classification was initially developed to predict prognosis and includes depth of tumor invasion into or beyond the wall of the tumors, invasion of or adherence to adjacent organs or structures (T), the number of regional lymph nodes involved (N), and presence or absence of distant metastasis (M) [3]. Since the mid-1980s, the TNM system has become our global “language of cancer” [4]. In many research studies, multiple clinicopathologic features are being investigated to determine the relationship with patient survival [5]–[7]. It is well accepted that T category is a significant or even independent predictor of survival in colorectal cancer [8], [9].

In clinical practice, there usually is another staging system called surgical TNM (sTNM), which is applied to stage and assess the cancer, and it potentially influences patient management guidelines [10], [11], [12]. The sTNM staging is also based on the tumors, lymph nodes, and metastasis but is defined by surgeons according to the intraoperative findings [10], [12], in contrast to TNM staging, which is performed by pathologists (pathologic TNM, pTNM). In addition to providing information on the cancer, intraoperative staging is utilized to allow for the selection of the optimal individualized surgical decision for the patient. It is commonly accepted that accurate staging is not only a foundation for deciding the most suitable subsequent therapy, but is also a critical tool for assessment of survival. It is important to obtain accurate intraoperative and postoperative staging, as these tools aid in the evaluation of the optimal extent of tumor resection and offer useful auxiliary treatment decisions. Nonetheless, in clinical practice, the assessments might exhibit differences between surgical and pathological stages of tumor depth [13], [14], [15]. Although some researchers have analyzed the sources and consequences of this phenomenon in patients with gastric cancer [12], the consequences of overtreatment or undertreatment due to inconsistent assessments have not been further investigated, as well as the impact on patient postoperative outcome. Currently, few reports have focused on this issue in a large sample of colorectal cancer patients. It remains unclear whether or not the unconformity of staging results influences the survival of patients with colorectal cancer.

Thus, in this study we aimed to evaluate survival of colorectal cancer patients with inconsistent assessments of tumor depth between surgical and pathological staging. We also assessed the feasibility of a new revised pT category (r-pT category), which integrates the surgical T (sT) category with pathological T (pT) category for prognostic assessment, and investigated whether it exhibits any improvement in predictive capabilities.

## Methods

### Participants

Clinical information on all colorectal cancer patients who underwent surgery at the Department of Surgical Oncology at the First Hospital of China Medical University from April 1994 to December 2007 was retrospectively collected and then reviewed and analyzed. Patients with any of the following criteria were excluded from this study: (i) patients who died in the immediate postoperative period (within 30 days), (ii) patients with multiple adenocarcinomas of colon and rectum, (iii) patients with synchronous or metachronous tumors, (iv) patients with distant metastasis, (v) patients who underwent neoadjuvant treatment, (vi) patients with incomplete pathological data entries, and (vii) patients who were lost to follow-up. After considering the above criteria, there were 948 colorectal cancer patients in our study. The clinicopathologic data utilized included age, gender, date of surgery, date of death (if applicable), cause of death, date of follow-up, location of the primary tumor, tumor size, histologic grade, venous invasion, lymphovascular invasion, depth of invasion, tumor deposits, number of lymph nodes retrieved, and number of lymph node metastases. The information was obtained through the medical records for all patients. Tumors originating from cecum to sigmoid colon were defined as colon cancer; tumors located in the rectum or rectosigmoid junction were considered as rectal cancer [16].

### Classification Methods

During the surgical procedure for colorectal cancer, the tumor of each patient was examined, and the final macroscopic depth of invasion was confirmed by all of the surgeons present during the operation after tumor exploration was complete [17]. In order to ensure the integrity of the tumor specimen, surgeons did not cut open the tumor to determine sT staging. The pathologists subsequently evaluated the postoperative tumor staging. Selecting the postoperative therapeutic option and evaluating the prognosis of patients were still based on the pT staging.Macroscopic assessment of tumor depth during surgery named sT staging was performed as follows: sT1 lesions were diagnosed when the lesion felt normal, and the assessment combined with preoperative auxiliary examination; sT2 lesions were diagnosed when the lesion felt mobile on the muscle layer of the colorectal wall; sT3 lesions were diagnosed when tumor did not invade through the serosa, and the lesion felt nodular on the serosal layer of the colorectal wall; sT4a lesions when serosal involvement were visible and sT4b lesions were directly invaded, or was adherent to other organs or structures [12].

### Pathological Procedures

All specimens were fixed in formalin, embedded in paraffin, and stained with hematoxylin and eosin. The sections of tumor were examined by two independent pathologists and confirmed by a third pathologist to arrive at the final diagnosis. Disagreements regarding the diagnosis were resolved by consensus on subsequent review of the slides, with all three pathologists present [18].

### Follow Up

Postoperative follow-up was completed for the entire study population in November 2008. The median and mean follow-up periods were 39.0 months and 50.5 months (range: 1.1–167.1 months), respectively.

### Ethics Statement

The study was approved by the Research Ethics Committee of China Medical University, China. Written informed consent was obtained from all patients prior to participation in this study.

### Statistical Analysis

Continuous data are presented as mean ± standard deviation (SD). Cancer-specific survival was analyzed using Kaplan-Meier survival curves, and comparisons were made using the log-rank test. For the purpose of our study, we proposed a novel category, r-pT, in which sT4b was included in the pT category, patient survival was then assessed and compared according to staging using the r-pT category. Multivariate analysis was performed using Cox’s proportional hazards model. Two-step multivariate analyses were applied to identify which category (the pT category in current TNM staging, and the r-pT category) had the greater potential to predict patient survival. The predictive value was also evaluated using Harrell’s C statistic: a higher C statistic indicates a more desirable model for predicting outcome [19], [20]. Statistical analyses and graphics were performed using PASW Statistics 18.0 software (SPSS, Inc., Somers, NY, USA) and STATA MP ver.10 (StataCorp LP, College Station, TX) statistical software. A value of P<0.05 was considered to be statistically significant.

## Results

Clinicopathological characteristics of 948 colorectal cancer patients are listed in Table 1. In our study, there were 551 (58.1%) males and 397 females (41.9%; ratio 1.4∶1) with a median age of 62.00 years (range 20–88 years). Among these patients, 475 patients (50.1%) suffered colon tumors and 473 patients (49.9%) suffered rectum tumors. Patients were classified according to the following sT category and the pT category in current TNM staging: 13 (1.37%) patients, 61 (6.43%) patients, 124 (13.08%) patients, 527 (55.59%) patients, and 223 (23.52%) patients were sT1, sT2, sT3, sT4a and sT4b, respectively; 12 (1.27%) patients, 164 (17.30%) patients, 651 (68.67%) patients, 99 (10.44%) patients, and 22 (2.32%) patients were pT1, pT2, pT3, pT4a and pT4b, respectively. Univariate analysis identified the sT category (P<0.001) and the pT category in the seventh edition of TNM staging (P<0.001) were significantly correlated with prognosis (Table 1).

**Table 1:** Table 1. Clinicopathologic features of 948 patients with colorectal cancers.

	n^a^	5-YSR^b^(%)	P value
Gender			0.620
Male	551	68.6	
Female	397	72.3	
Age			0.110
≤60	433	73.3	
>60	515	67.1	
Tumor location			0.599
Colon	475	71.4	
Rectum	473	69.0	
Size			0.662
≤5.0 cm	543	71.2	
>5.0 cm	405	69.2	
Venous invasion			<0.001
Positive	9	16.7	
Negative	939	70.8	
Lymphovascular invasion			<0.001
Positive	58	43.0	
Negative	890	72.0	
Histologic grade			0.001
Well	435	75.5	
Moderate	435	66.3	
Poor	78	57.3	
Tumor deposits			<0.001
Positive	135	36.9	
Negative	813	75.5	
pT category			<0.001
pT1	12	100.0	
pT2	164	83.4	
pT3	651	71.7	
pT4a	99	47.5	
pT4b	22	35.8	
sT category			<0.001
sT1	13	100.0	
sT2	61	77.5	
sT3	124	68.1	
sT4a	527	74.9	
sT4b	223	53.5	
pN category			<0.001
pN0	561	84.4	
pN1	271	58.7	
pN2	116	25.2	

n^a^: Number of patients.

5-YSR^b^: 5-year accumulative survival rate.

**Figure 1:**
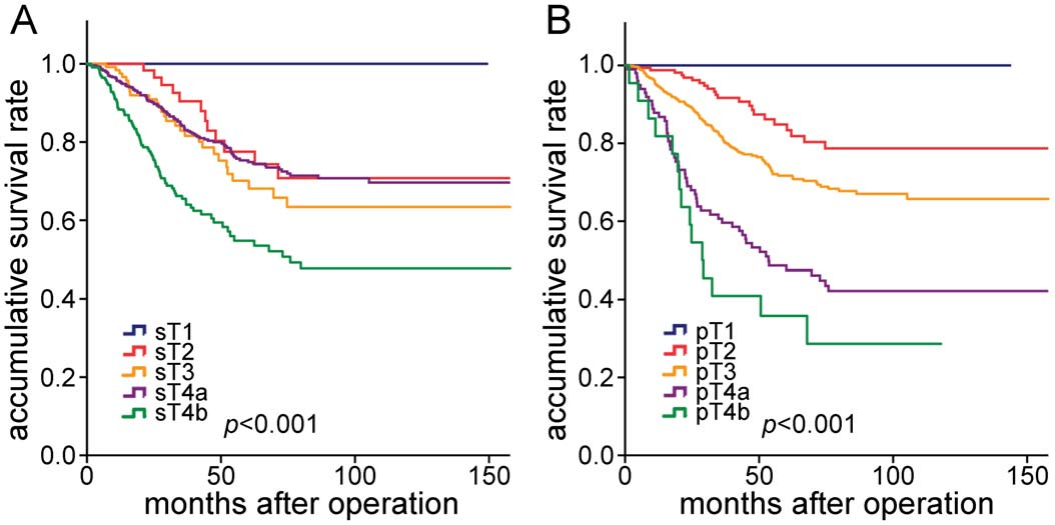
Comparison of survival curves among the patients according to the sT and pT category. **A,** Survival curves of patients with different sT categories. **B,** Survival curves of patients with different pT categories.

The 5-year survival rates for all patients stratified according to sT category were calculated for each group. The 5-year survival rate for patients with sT4b cancer was significantly lower compared to sT3 cancer patients (53.5% vs. 68.1%; P = 0.002), and also lower compared to sT4a cancers (53.5% vs. 74.9%; P<0.001), however, sT3 and sT4a cancers had similar 5-year survival rates (68.1% vs. 74.9%; P = 0.404) (Table 1, Figure 1A). When prognosis was compared, there were significant differences among pT subcategories (Figure 1B).

**Figure 2:**
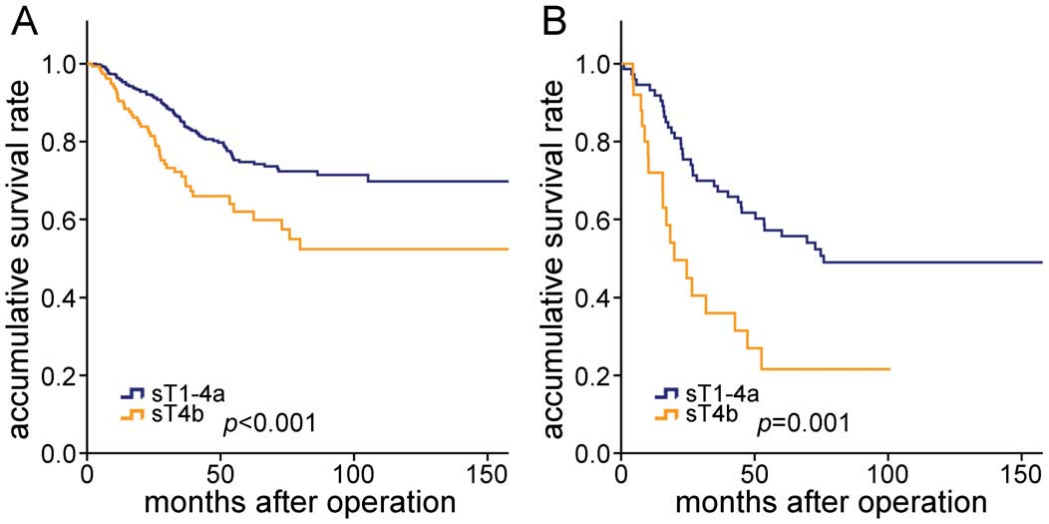
Comparison of survival curves of the patients stratified by pT and sT category. **A,** For patients in pT3 category, there was prognostic difference between sT4a and sT4b cancer (P<0.001). **B,** For patients in pT4a category, there was prognostic difference between sT4a and sT4b cancer (P = 0.001).

For patients in each pT category, prognosis was compared according to the sT category, and no significant differences were found among sT1, sT2, sT3, and sT4a in pT3 category, as well as in pT4a category (P>0.05). As shown in Figure 2, for patients in pT3 category, there was a significant prognostic difference between sT1-4a and sT4b cancers (P<0.001) (Figure 2A), and for patients in pT4a category, there was a significant prognostic difference between sT1-4a and sT4b cancers (P = 0.001) (Figure 2B). Therefore, marked prognostic heterogeneity was demonstrated in the pT3 and pT4a categories.

**Figure 3:**
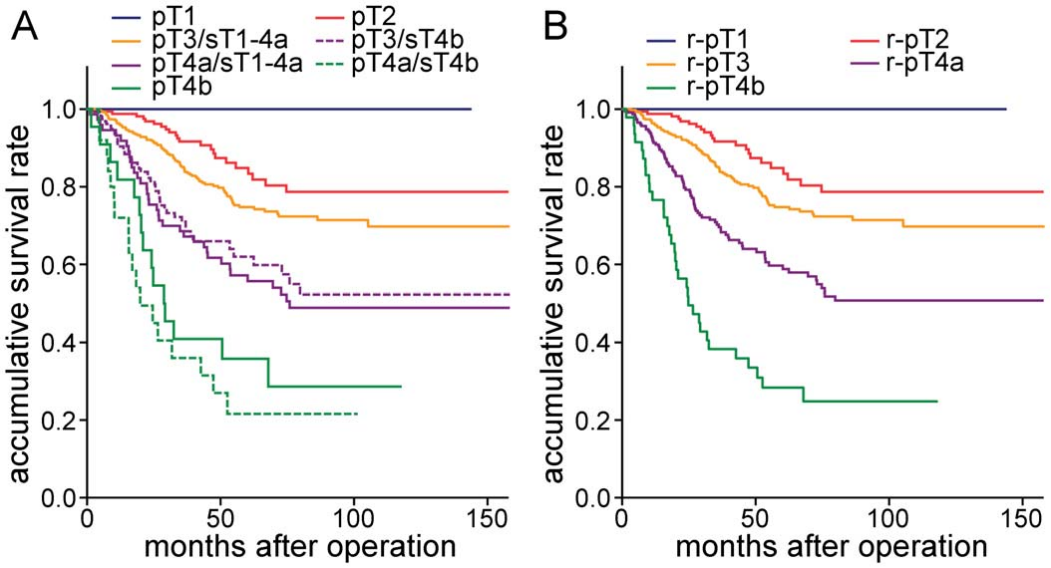
Survival curves of patients with colorectal cancers according to the r-pT category. **A,** Survival curves of patients grouped by pT categories when patients in pT3 and pT4a were stratified by sT categories. There were no significant differences between pT3/sT4b and pT4a/sT1-4a (P = 0.599), as well as pT4a/sT4b and pT4b (P = 0.351). **B,** Survival curves of patients stratified by r-pT category, there were significant differences among the patients.

We then integrated sT4b with the pT category and reclassified patients in the pT3 and pT4a categories. We compared the prognosis, and no significant differences were found between pT3/sT4b and pT4a/sT1-4a (P = 0.599), as well as pT4a/sT4b and pT4b (P = 0.351), which suggests that the heterogeneity disappeared among these groups (Figure 3A). Therefore, we incorporated pT3/sT4b into pT4a/sT1-4a, as well as pT4a/sT4b into pT4b. We then compared survival curves, and found significant differences among the different categories (Figure 3B). Based on these results, we proposed a novel category, r-pT, in which patients categorized as pT3 with sT4b were incorporated into the category pT4a (r-pT4a), and patients categorized as pT4a with sT4b were incorporated into the category pT4b (r-pT4b).

To further elucidate the correlation between r-pT category and prognosis, two-step multivariate analyses was used. In the step one multivariate analysis, pN category, lymphovascular invasion, sT category, pT category and tumor deposits were confirmed to be independent prognostic factors (P = 0.001 for lymphovascular invasion, P = 0.041 for sT category, P<0.001 for all the others, Table 3). Interestingly, in the step two multivariate analysis, in which the r-pT category was also considered together with the factors of the step one multivariate analysis, pN category, lymphovascular invasion and tumor deposits remained significant (P = 0.002 for lymphovascular invasion, P<0.001 for all others, Table 3). In the step two multivariate analysis, the pT category lost its significance and was substituted by the r-pT category.

**Table 3:** Two-step multivariate analysis of the prognostic factors for 948 patients.

	Relative Risk	95% Confidence Interval	*P* value*
Step 1†			
sT category	1.232	1.009–1.504	0.041
pT category	1.693	1.393–2.059	<0.001
pN category	2.303	1.923–2.759	<0.001
Tumor deposits	1.821	1.347–2.463	<0.001
Lymphovascular invasion	1.927	1.288–2.885	0.001
Step 2‡			
pN category	2.283	1.906–2.735	<0.001
Lymphovascular invasion	1.902	1.270–2.849	0.002
Tumor deposits	1.833	1.354–2.480	<0.001
r-pT category	1.846	1.566–2.176	<0.001

* *P* values were made by Cox’s proportional hazards model.

† Step 1, with consideration of all significantly important prognostic factors in univariate analysis, except for the r-pT category.

‡ Step 2, with consideration of all significantly important prognostic factors in univariate analysis, including the r-pT category.

The r-pT and pT categories were measured by Harrell’s C statistic to determine which exhibited a superior predictive capacity. Our findings demonstrated that the r-pT category (Harrell’s C = 0.668; 95% CI:0.635–0.702) was superior to the pT category in current TNM staging (Harrell’s C = 0.636; 95% CI:0.604–0.667) (P = 0.002).

## Discussion

The UICC/AJCC TNM staging system, although controversial, is considered the most powerful and reliable predictor of prognosis for colorectal cancer globally. Presently, it is generally accepted that the depth of tumor invasion in the TNM staging system is an important prognostic factor. In particular, pT4 as a complex subgroup is strongly correlated with adverse events [21]. In the new era of comprehensive diagnostic modalities, the importance of surgical staging in standard cancer management has been well established [11]. In many clinical settings, defining the patient prognosis and subsequent therapeutic management is potentially difficult in the absence of appropriate surgical staging [11]. For example, in a report by Gajra *et al*, they emphasized that surgical staging of cancer impacts the prognosis of non-small-cell lung cancer [22]. In addition, the relationship between intraoperative and postoperative staging is of potentially great importance [11]. For cases in which accurate macroscopic and pathologic assessments were obtained, it is possible to provide a much more reasonable estimate of prognosis. However, in clinical practice, intraoperative and postoperative assessments of T category frequently have unconformity due to lots of reasons, resulting in inadequate tumor staging and posing an obstacle to standardized treatment management. The unconformity of gastric cancer staged by pT and sT staging has been previously reported in numerous studies [12], [13]. In this study, we present a retrospective single-center analysis of 948 Chinese patients with colorectal cancer. There were 755 patients with inconsistent staging results in our study, and our findings demonstrated a noticeable tendency in which surgeons stage tumors in a low category to a higher category during the surgical procedure compared to the pathological staging (Table 2).

**Table 2:** Comparison and kappa statistics between the sT and pT categories patients.

		sT					n^a^
		sT1	sT2	sT3	sT4a	sT4b	
pT	sT1	5	6	1	0	0	12
	pT2	6	32	36	69	21	164
	pT3	2	20	76	396	157	651
	pT4a	0	3	11	60	25	99
	pT4b	0	0	0	2	20	22
n^a^		13	61	124	527	224	949

n^a^: Number of patients.

In several previous studies concerning gastric cancer, some researchers only explained the possible reasons of this unconformity, but the patients’ prognostic outcomes that were influenced by the unconformity were not further investigated [12], [13]. Until now, the data regarding colorectal cancer and this issue has been limited. In our study, using univariate analysis, we found that the sT category was an important independent prognostic factor. Simultaneously, the cancer-specific survival rates of patients stratified by sT category were compared among the different pT groups. We found that there was a significant difference between sT1-4a and sT4b in pT3 cancers (P<0.001), as well as in pT4a cancers (P = 0.001). Our findings indicated that there was prognostic heterogeneity in these groups. Taken together, our findings indicated that there are potential shortcomings in the current pT category for staging patients when their surgical and pathological results are inconsistent, and sT4b cancers should not be neglected when colorectal tumors were classified according to the pT category.

We compared the 5-year survival curves of the patients categorized as having pT3 and pT4 cancers. We found that there were no significant prognostic differences between pT3/sT4b and pT4a/sT1-4a, as well as pT4a/sT4b and pT4b. These loss of differences indicated that pT3/sT4b cancers might more homogeneous with pT4a/sT1-4a cancers, as well as pT4a/sT4b with pT4b cancers. This suggested that subclassification of pT3/sT4b and pT4a/sT1-4a cancers into one group is warranted, as well as pT4a/sT4b and pT4b cancers should be subclassified into one group. We compared survival curves, and found significant differences among the different categories. Based on above results, we proposed a novel r-pT category: patients in pT3 with sT4b cancers were categorized as r-pT4a, and patients in pT4a with sT4b cancers were categorized as r-pT4b. And then, we tested this novel r-pT category in our study. When comparing the prognostic power of the r-pT category to that of the present pT category, 2-step multivariate analysis was utilized. In the step 1 multivariate analysis, the pT category was identified as an independent prognostic factor, as well as the sT category. However, when the step 2 multivariate analysis was applied, the pT category and sT category lost significance and were substituted by the r-pT category. This result suggests that the r-pT category had superior prognostic value compared to the pT category. In addition, we used Harrell’s C statistic to further elucidate whether the r-pT category was superior to pT category in terms of predictive capacity, and the results demonstrated that the r-pT category stage exhibited a stronger predictive power. Both statistic methods confirmed that the novel r-pT category was more accurate than the pT category in prognostic assessment.

It is commonly accepted that intraoperative assessment of tumor depth is often difficult. Nonetheless, sT4b, a category that represents tumors that directly invade other organs, is much easier for surgeons to distinguish and identify compared to other subgroups during surgery. Based on these considerations, this novel category which incorporated the sT4b category into the pT category was simple to perform in clinical settings.

We acknowledge that there are several limitations in this study. Our study is based on the retrospective analysis of a mono-institutional clinicopathological database of 948 Chinese colorectal cancer patients. Certainly, our conclusions are constrained by the usual limitations of retrospective analysis from a single institution. Whether our results can be applied to other institutions remains to be demonstrated. We look forward to performing studies with a larger sample size, as well as international multicentric studies in patients with colorectal cancer in the near future and authenticating the accuracy in a large population-based collective of patients.

According to the results generated in our study, we suggest that macroscopic tumor invasion of adjacent organs should be taken into account for prognosis of patients with colorectal cancer. When patients are categorized as pT3 with sT4b, they could be reclassified as r-pT4a, and when patients are categorized as pT4a with sT4b, they could be reclassified as r-pT4b. This novel r-pT category that we proposed could be applied to predict the patient prognosis and is also potentially superior to the seventh edition of the T category for assessment of the prognostic power in colorectal cancer.

## References

[1] Ostadi MA, Harnish JL, Stegienko S, Urbach DR (2007) Factors affecting the number of lymph nodes retrieved in colorectal cancer specimens. Surg Endosc 21: 2142–2146. 17522917 10.1007/s00464-007-9414-6

[2] Jemal A, Siegel R, Ward E, Hao Y, Xu J, et al (2008) Cancer Statistics, 2008. CA Cancer J Clin 58: 71–96. 18287387 10.3322/CA.2007.0010

[3] Sobin LH, Gospodarowicz MK, Wittekind CH (2009) UICC TNM classification on malignant tumours. 7th edition.

[4] Greene FL, Sobin LH (2009) A worldwide approach to the TNM staging system: collaborative efforts of the AJCC and UICC. J Surg Oncol 99: 269–272. 19170124 10.1002/jso.21237

[5] Tong LL, Gao P, Wang ZN, Song YX, Xu YY, et al (2011) Can lymph node ratio take the place of pN categories in the UICC/AJCC TNM classification system for colorectal cancer? Ann Surg Oncol 18: 2453–2460. 21455596 10.1245/s10434-011-1687-2

[6] Nagtegaal ID, Quirke P (2007) Colorectal tumour deposits in the mesorectum and pericolon: a critical review. Histopathology 51: 141–149. 17532768 10.1111/j.1365-2559.2007.02720.x

[7] Tong LL, Gao P, Wang ZN, Yue ZY, Song YX, et al (2011) Is pT2 subclassification feasible to predict outcome in colorectal cancer ? Ann Surg Oncol 18: 1389–1396. 21107740 10.1245/s10434-010-1440-2

[8] Hemminki K, Santi I, Weires M, Thomsen H, Sundquist J, et al (2010) Tumor location and patient characteristics of colon and rectal adenocarcinomas in relation to survival and TNM classes. BMC Cancer 10: 688. 21176147 10.1186/1471-2407-10-688 PMC3022888

[9] Goldstein NS, Turner JR (2000) Pericolonic tumour deposits in patients with T3N+M0 colon adenocarcinomas: markers of reduced disease free survival and intra-abdominal metastases and their implications for TNM classification. Cancer 88: 2228–2238. 10820343

[10] Harisi R, Schaff Z, Flautner L, Winternitz T, Jaray B, et al (2008) Evaluation and comparison of the clinical, surgical and pathological TNM staging of colorectal cancer. Hepatogastroenterology 55: 66–72. 18507081

[11] Markman M (2003) Surgical staging of cancer: impact on prognosis and potential for bias in clinical trials. Curr Onco Rep 5: 437–438. 10.1007/s11912-003-0001-2 14521801

[12] Madden MV, Price SK, Learmonth GM, Dent DM (1987) Surgical staging of gastric carcinoma: sources and consequences of error. Br J Surg 74: 119–121. 3815027 10.1002/bjs.1800740217

[13] Xu HM, Chen JQ, Wang SB, Qi CL, Shan JX (1995) Prediction of the staging and biological behavior from the serosal change in gastric cancer. J China Medical University 24: 352–355.

[14] Chen YG, Liu YL, Jiang SX, Wang XS (2011) Adhesion pattern and prognosis studies of T4N0M0 colorectal cancer following en bloc multivisceral resection: evaluation of T4 subclassification. Cell Biochem Biophys 59: 1–6. 20740326 10.1007/s12013-010-9106-z

[15] Compton CC, Greene FL (2004) The staging of colorectal cancer: 2004 and beyond. CA Cancer J Clin 54: 295–308. 15537574 10.3322/canjclin.54.6.295

[16] Chok KS, Law WL (2007) Prognostic factors affecting survival and recurrence of patients with pT1 and pT2 colorectal cancer. World J Surg 31: 1485–1490. 17510767 10.1007/s00268-007-9089-0

[17] Chen JQ, Qi CL, Wang SB, Shan JX, Zhang WF, et al (1986) The serosal types of gastric cancer and its biologic significance. J China Medical University 15: 347–350.

[18] Sun Z, Zhu GL, Lu C, Guo PT, Huang BJ, et al (2009) A novel subclassification of pT2 gastric cancers according to the depth of muscularis propria invasion: superficial muscularis propria versus deep muscularis propria/subserosa. Ann Surg 249: 768–775. 19387327 10.1097/SLA.0b013e3181a3df77

[19] Harrell FE Jr, Lee KL, Mark DB (1996) Multivariable prognostic models: issues in developing models, evaluating assumptions and adequacy, and measuring and reducing errors. Stat Med 15: 361–387. 8668867 10.1002/(SICI)1097-0258(19960229)15:4<361::AID-SIM168>3.0.CO;2-4

[20] Harrell FE Jr, Lee KL, Califf RM, Pryor DB, Rosati RA (1984) Regression modelling strategies for improved prognostic prediction. Stat Med 3: 143–152. 6463451 10.1002/sim.4780030207

[21] Tong LL, Gao P, Wang ZN, Song YX, Xu YY, et al (2012) Is the 7th edition of the UICC/AJCC TNM staging system reasonable for patients with tumor deposits in colorectal cancer? Ann Surg 225: 208–213. 10.1097/SLA.0b013e31821ad8a2 21527844

[22] Gajra A, Newman N, Gamble GP, Kohman LJ, Graziano SL (2003) Effect of number of lymph nodes sampled on outcome in patients with stage I non-small-cell lung cancer. J Clin Oncol 21: 1029–1034. 12637467 10.1200/JCO.2003.07.010

